# An IPSO-FW-WSVM Method for Stock Trading Signal Forecasting

**DOI:** 10.3390/e25020279

**Published:** 2023-02-02

**Authors:** Yingjun Chen, Zhigang Zhu

**Affiliations:** 1 Department of Computer Science and Technology, Tongji University, Shanghai 201804, China; 2 School of Health Science and Engineering, University of Shanghai for Science and Technology, Shanghai 200093, China

**Keywords:** improved particle swarm optimization (IPSO), feature-weighted support vector machine (FW-WSVM), trading signals forecasting

## Abstract

Trading signal detection is a very popular yet challenging research topic in the financial investment area. This paper develops a novel method integrating piecewise linear representation (PLR), improved particle swarm optimization (IPSO) and a feature-weighted support vector machine (FW-WSVM) to analyze the nonlinear relationships between trading signals and the stock data hidden in historical data. First, PLR is applied to generate numerous trading points (valleys or peaks) based on the historical data. These turning points’ prediction is formulated as a three-class classification problem. Then, IPSO is utilized to find the optimal parameters of FW-WSVM. Lastly, we conduct a series of comparative experiments between IPSO-FW-WSVM and PLR-ANN on 25 stocks with 2 different investment strategies. The experiment results show that our proposed method achieves higher prediction accuracy and profitability, which indicates the IPSO-FW-WSVM method is effective in the prediction of trading signals.

## 1. Introduction

Designing and implementing various predictive techniques for complex data analysis has attracted great scientific interest [1,2]. Among the research, stock trading point prediction has been an attractive yet challenging research topic. Therefore, many investors and researchers have conducted plenty of research in this field. However, the stock market is a nonlinear, changeable and complex system affected by many factors such as government policies, international situations, economic environments, interest rates and market capitalization. Despite the volatility of the stock market, researchers are still trying to develop more effective prediction techniques due to the benefits involved in accurate predictions.

With regard to the techniques used to find optimal trading points, some of them are based on financial analysis, and others are artificial intelligence methods. Financial analysis usually uses two main analysis methods, which are fundamental and technical analysis [3,4]. Fundamental analysis uses macroeconomic, industrial and business indicators to predict trend reversals [5,6,7]. Technical analysis assumes that past behavior has an impact on price evolution and trading decisions are made based on historical prices and some useful technical indicators such as moving averages and the relative strength index [8,9,10]. Financial time series data are inherently dynamic, nonlinear [11,12] and do not follow the fixed patterns. Therefore, making the right trading decisions is very difficult through financial analysis. In contrast, artificial intelligence algorithms excel at handling dynamic and nonlinear data in financial markets and are widely applied to predict trading points.

Over the past few decades, many artificial intelligence and machine learning algorithms have been developed as major tools in the financial investment field, such as artificial neural networks [13,14,15], support vector machines [16,17,18,19], rough set theory [20,21,22,23], Bayesian analysis [24,25,26,27] and evolutionary learning algorithms [28,29,30,31]. However, most past studies have focused on the accurate prediction of stock prices rather than trading decisions, since it is more difficult to predict the buying and selling points of stocks than to predict the changes in stock prices.

In recent years, artificial neural networks have been widely used to predict the turning points of a stock. For example, Chiang et al. proposed an adaptive intelligent stock trading decision support system using a particle swarm algorithm and neural network to predict the future movement direction [32]. This method overcame the weakness of the selection of inputs and parameter settings in the traditional ANN approach. However, artificial neural networks have many disadvantages, such as black box technology, easy overfitting, large computational complexity, slow convergence speed and easily falling into local minima. To overcome these disadvantages, the SVM [33] has attracted much attention. It has become a very popular research method in the financial investment field. Luo et al. conducted a set of improvements to the PLR-WSVM by adding some relative indicators with more valuable information into the input variables [34], simplifying the turning point prediction into a two-class problem, adapting the threshold values of PLR and using the WSVM to find the stock trading signals. Chen and Hao proposed a novel method, PLR-FW-WSVM, to predict trading points [35]. Despite the SVM having good generalization performance, whether the parameters are appropriate often affects its classification performance and the generalization ability [36,37]. The traditional grid search algorithm has low efficiency and a large amount of calculations, in addition to being time-consuming and having an unsatisfactory effect [38]. PSO not only has the global optimization ability but also has an efficient convergence ability and strong local optimization ability [39]. The optimal solution can be found through coordination and information exchange among individuals. In order to find the optimal solution to the FW-WSVM model, this paper proposes improved particle swarm optimization (IPSO) and integrates it into FW-WSVM to find the trading signals in the financial field (IPSO-FW-WSVM). First, the turning points are generated by a PLR algorithm. Secondly, the information gain is calculated in order to set the weight of each feature. Lastly, we utilize IPSO to optimize the FW-WSVM model parameters for stock trading decision.

The rest of this paper is organized as follows. In Section 2, we briefly present the theories of PLR, the FW-WSVM based on the information gain and FW-WSVM parameter optimization based on IPSO. In Section 3, we present the research design, which contains input variable selection, data labeling and performance measuring. Section 4 presents some experimental results to validate the performance of our proposed method. Section 5 gives a summary of this work and some brief future work.

## 2. Methodology

**Figure 1:**
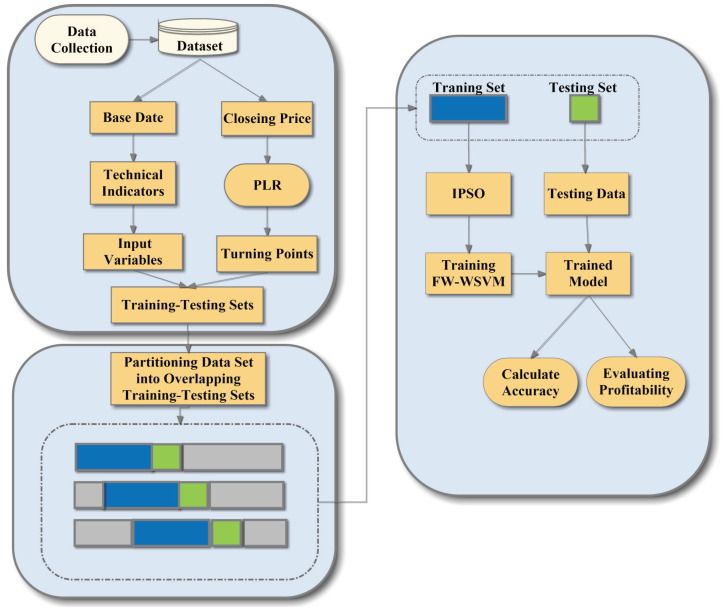
The flow chart of the proposed framework.

This paper utilizes the IPSO-FW-WSVM method to detect trading signals in financial time series data. PLR first generates trading points from the historical stock price database. Secondly, the whole dataset is divided into a lot of overlapping training-testing sets, which can reduce the time-varying characteristics of stock data. Thirdly, the IPSO-FW-WSVM model is applied to learn the relationship between the input features and trading signals. Finally, the trained model is adopted to compute the accuracy of the model and evaluate the profitability with two investment strategies. The flow chart of the proposed framework is shown in Figure 1.

### 2.1. PLR

**Figure 2:**
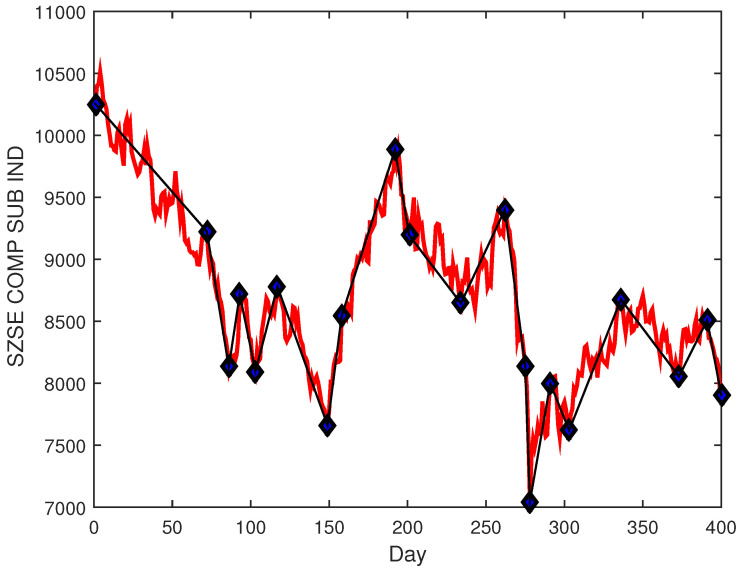
The segments generated by PLR.

PLR was developed for pattern matching, and it can be used to generate turning points in financial time series data. Let $X=\{x_{1},x_{2},\ldots ,x_{N}\}$ denote the financial time series data. *X* can be divided into *M* segments, which are expressed as follows:

(1) $$S_{PLR}=\left\{L_{1}\left(x_{1},x_{2},\ldots ,x_{t_{1}}\right),L_{2}\left(x_{t_{1}+1},x_{t_{1}+2},\ldots ,x_{t_{2}}\right),\ldots ,L_{M}\left(x_{t_{M-1}+1},x_{t_{M-1}+2},\ldots ,x_{t_{M}}\right)\right\}$$

where $t_{i}\left(i=1,2,\ldots ,M\right)$ is the end of the ith segment. Therefore, $S_{PLR}$ provides a lot of segments belonging to an uptrend or a downtrend with low and high price points, as shown in Figure 2.

**Figure 3:**
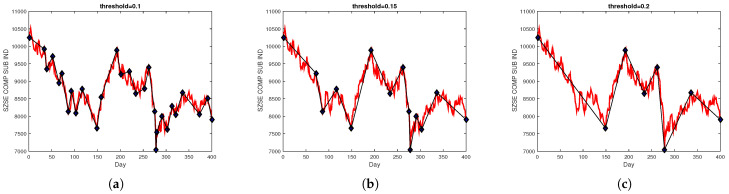
The turning points obtained using different thresholds. (**a**) Results for PLR (threshold = 0.1). (**b**) Results for PLR (threshold = 0.15). (**c**) Results for PLR (threshold = 0.2).

The threshold of segment representation is a significant parameter affecting the results of PLR. Figure 3 shows some different segmentation graphs based on different threshold values for the Shenzhen Stock Exchange Component Index (SZSE COMP SUB IND). As shown in Figure 3, the higher threshold value created longer trend patterns and generated only a few segments, while a smaller threshold generated a lot of segments. Therefore, it should be more reasonable to specify different thresholds for different price fluctuations. In this paper, we utilized the SD algorithm [34] to automatically select the threshold, and different thresholds for different price fluctuations were calculated according to a percentage of turning points in the parameter pct.

### 2.2. FW-WSVM Based on the Information Gain

In this section, a brief introduction for the FW-WSVM method based on information gain is given, and more details can be found in [35]. Let $T_{train}=\left\{\left(x_{1},y_{1}\right),\left(x_{2},y_{2}\right),\ldots ,\left(x_{N},y_{N}\right)\right\}$ be the training dataset, where $x_{i}=\left(x_{i}^{1},x_{i}^{2},\ldots ,x_{i}^{n}\right)\in X=R^{n}$ denotes the ith (i=1,2,…N) input vector, *n* is the size of vector and $y_{i}\in Y=\left\{-1,+1\right\}$ denotes the label. The dataset $T_{train}$ is separated into two classes through the hyperplane ω·ϕ(xP)+b=0, where *P* is a n×n feature-weighted matrix. The classifier should satisfy the following conditions:

(2) $$y_{i}\left(\omega \cdot \phi \left(xP\right)+b\right)\geq 1-\zeta_{i},\left(\zeta_{i}\geq 0,i=1,2,\ldots ,N\right)$$

where $\phi :R^{n}\to R^{m}$ is used to map the input space $R^{n}$ to the high-dimensional feature space $R^{m}$ and $\zeta_{i}$ is the slack variable. Therefore, The objective optimization can be expressed as follows:

(3) $$\begin{array}{cc} \min_{\omega ,b,\zeta } & :J\left(\omega ,\zeta \right)=\frac{1}{2}{\left||\omega |\right|}^{2}+\sum_{i=1}^{N}C_{i}\zeta_{i}\\ \; & \mathrm{subject}\;\mathrm{to}:\;\left\{\begin{array}{c} y_{i}\left(\omega \cdot \phi \left(xP\right)+b\right)\geq 1-\zeta_{i},i=1,2,\ldots ,N\\ \zeta_{i}\geq 0,i=1,2,\ldots ,N \end{array}\right. \end{array}$$

where $C_{i}$ is the weighted constant parameter, which is defined as follows:

(4) $$C_{i}=v_{i}C$$

where $v_{i}$ is the weight of the instance $x_{i}$. Thus, the corresponding Lagrangian function is Equation (5):

(5) $$L_{p}\left(\omega ,b,\zeta ,\alpha ,\mu \right)=\frac{1}{2}{\left||\omega |\right|}^{2}+\sum_{i=1}^{N}C_{i}\zeta_{i}-\sum_{i=1}^{N}\alpha_{i}\left[y_{i}\left(\omega \cdot \phi \left(x_{i}P\right)+b\right)-1+\zeta_{i}\right]-\sum_{i=1}^{N}\mu_{i}\zeta_{i}$$

where $\alpha_{i}$ and $\mu_{i}$ are the non-negative Lagrange multipliers. Therefore, the dual problem can be expressed as follows:

(6) $$\begin{array}{cc} \max_{\alpha } & :Q\left(\alpha \right)=\sum_{i=1}^{N}\alpha_{i}-\frac{1}{2}\sum_{i=1}^{N}\sum_{j=1}^{N}\alpha_{i}\alpha_{j}y_{i}y_{j}\phi \left(x_{i}P\right)\phi \left(x_{j}P\right)\\ \; & \mathrm{subject}\;\mathrm{to}:\;\left\{\begin{array}{c} \sum_{i=1}^{N}\alpha_{i}y_{i}=0,i=1,2,\ldots ,N\\ 0\leq \alpha_{i}\leq C_{i},i=1,2,\ldots ,N \end{array}\right. \end{array}$$

The decision function of the classification problem can be obtained with Equation (7):

(7) $$f\left(x\right)=sign(\sum_{i=1}^{N}\alpha_{i}y_{i}K\left(x_{i}P,x_{j}P\right)+\frac{1}{N_{S}}\sum_{0<\alpha_{j}<C_{i}}\left[y_{j}-\sum_{i=1}^{N}\alpha_{i}y_{i}K\left(x_{i}P,x_{j}P\right)\right])$$

where $N_{S}$ is the number of the support vectors and *P* is the feature-weighted matrix.

How to obtain the weighted matrix *P* is the key to the FW-WSVM algorithm. In this research, information gain is used to measure the weighted matrix *P*. Let $|C_{\left\{i,T_{train}\right\}}|$ be the size of the dataset $C_{\left\{i,T_{train}\right\}}$. The probability of a sample belonging to class $C_{+1}$/$C_{-1}$ can be approximately calculated through $|C_{\left\{i,T_{train}\right\}}|/N$. The expected information is as follows:

(8) $$Info\left(T_{train}\right)=-\sum_{i\in \left\{-1,+1\right\}}\frac{|C_{\left\{i,T_{train}\right\}}|}{N}\log\left(\frac{|C_{\left\{i,T_{train}\right\}}|}{N}\right)$$

Let $F_{feature}$ be the feature selected to split the set $T_{train}$, and let $F_{feature}$ have *v* different values. It can produce *v* subsets, indicated by $D_{1},D_{2},\ldots ,D_{v}$. Then, the expected information is as follows:

(9) $$\begin{array}{cc} Info_{F}\left(T_{train}\right) & =\sum_{j=1}^{v}\frac{|D_{j}|}{N}Info\left(D_{j}\right)=-\sum_{j=1}^{v}\frac{|D_{j}|}{N}\sum_{i\in \left\{-1,+1\right\}}\frac{|C_{\left\{i,D_{j}\right\}}|}{|D_{j}|}\log\left(\frac{|C_{\left\{i,D_{j}\right\}}|}{|D_{j}|}\right) \end{array}$$

where $|D_{j}|$ is the size of the set $D_{j}$, $|C_{\left\{i,D_{j}\right\}}|$ is the size of samples belonging to the class $C_{+1}$/$C_{-1}$ in set $D_{j}$ and i=−1,+1,j=1,2,…,v. Thus, the information gain is as follows:

(10) $$InfoGain\left(F\right)=\sqrt[{2}]{Info\left(T_{train}\right)-Info_{F}\left(T_{train}\right)}$$

If the information gain is greater, then the corresponding feature is more important, and the contribution to classification is greater. Consequently, the feature-weighted matrix *P* is as follows:

(11) $$P=\left\{\begin{array}{cccc} InfoGain(f_{1}) & 0 & \cdots & 0\\ 0 & InfoGain(f_{2}) & 0 & \vdots \\ \vdots & 0 & \ddots & 0\\ 0 & \cdots & 0 & InfoGain(f_{n}) \end{array}\right\}$$

where $InfoGain(f_{i})$ describes the weight of each feature and i=1,2,…,n.

### 2.3. FW-WSVM Parameter Optimization Based on IPSO

In the FW-WSVM algorithm, the value of factor *C* and the value of δ in the kernel function can obviously affect the performance of the system. It is difficult to choose the two important optimal parameters (*C* and δ) by virtue of expert experience. In order to find the optimal values for these parameters with the smallest generalization error, PSO is used to optimize the parameter selection of the FW-WSVM model.

The PSO algorithm is a heuristic search algorithm which was derived from the flocking behavior of insects, herds of animals, flocks of birds, schools of fish, etc. The algorithm searches the solution space of the problem by simulating the foraging behavior of birds. In the PSO algorithm, each particle moves at a certain speed in the search space, changes its position according to the fitness value in the environment and shares the information with other particles.

Let the total number of particles be *n*, the dimension be *d*, the position of the *i* particle be $x_{i}=\left(x_{i1},x_{i2},\ldots ,x_{id}\right)$ and the corresponding velocity be $v_{i}=\left(v_{i1},v_{i2},\ldots ,v_{id}\right)$, where i=1,2,…,n. In each iteration, the particle updates its position by tracking two optimal solutions. The first one is the individual historical optimal solution, indicated as pbest, where $p_{i}=\left(p_{i1},p_{i2},\ldots ,p_{id}\right)$, and the other is the global optimal solution, indicated as gbest, where $p_{g}=\left(p_{g1},p_{g2},\ldots ,p_{gd}\right)$. The particle updates its velocity and position as follows:

(12) $$v_{i}^{k+1}=\omega_{k}\cdot v_{i}^{k}+c_{1k}\cdot \gamma_{1}\cdot \left(p_{i}^{k}-x_{i}^{k}\right)+c_{2k}\cdot \gamma_{2}\cdot \left(p_{g}^{k}-x_{i}^{k}\right)$$

(13) $$x_{i}^{k+1}=x_{i}^{k}+\beta_{k}\cdot v_{i}^{k+1}$$

where $\omega_{k}$ is the inertia weight, $c_{1k}$ and $c_{2k}$ are the local search ability and global search ability learning factors, respectively, $\gamma_{1}$ and $\gamma_{2}$ represent random numbers between [0,1] and $\beta_{k}$ is the factor to speed up the convergence speed of the algorithm when updating the positions of particles:

(14) $$\beta_{k}=1-\tan\left(\frac{\pi }{4}\cdot \frac{k}{T_{\max}}\right)$$

where $T_{\max}$ is the maximum evolutionary quantity.

The setting of the inertia weight has a great influence on the convergence speed of the algorithm. A larger inertia weight has a stronger global search ability for jumping out of the local optimal solution, and a smaller inertia weight is conducive to local searches, increasing the convergence speed of the algorithm. The inertia weight has a great influence on the performance of the algorithm, and there have been many studies on it [40,41,42,43]. The more successful one is the linearly adjusted inertia weight particle swarm algorithm [44]. The idea is that the inertia weight decreases linearly with the increase in the number of iterations of the algorithm. The actual search process of the PSO algorithm is nonlinear and highly complex, and the strategy of linearly decreasing the inertia weight often cannot reflect the actual optimal search process. In order to overcome the deficiency of a linearly decreasing weight, this paper proposes a non-linear decreasing strategy:

(15) $$\omega_{k}=\omega_{max}-\left(\omega_{max}-\omega_{min}\right)\cdot \exp\left(1-\frac{T_{\max}}{k}\right)$$

where $\omega_{max}$ and $\omega_{min}$ are the maximum and minimum of the inertial weight, respectively, and $\omega_{k}$ is expressed in the inertia weight value of the *k*th iteration.

In this paper, we control the better global searching and local searching of particles by changing the learning factors $c_{1k}$ and $c_{2k}$ [45]. In the early stage of a search, a larger cognition coefficient $c_{1k}$ and a smaller social coefficient $c_{2k}$ are set so individual cognition occupies a dominant position, and the particles can develop new search areas in a larger search space. In the later stage, a larger social coefficient and a smaller cognitive coefficient are set. The updated formulas of $c_{1k}$ and $c_{2k}$ are as follows:

(16) $$c_{1k}=c_{1\min}+\left(c_{1\max}-c_{1\min}\right)\cdot \frac{T_{\max}-k}{T_{\max}}$$

(17) $$c_{2k}=c_{2\min}+\left(c_{2\max}-c_{2\min}\right)\cdot \frac{k}{T_{\max}}$$

The flow of optimization of the FW-WSVM parameters using IPSO is illustrated in Algorithm 1.    
**Algorithm 1:** Description of FW-WSVM parameter optimization based on IPSO***Step 1***: Determine the range of *C* and δ in FW-WSVM.***Step 2***: Initialize IPSO algorithm parameters $T_{\max}$, $\omega_{min}$, $\omega_{max}$, $c_{1\min}$, $c_{1\max}$, $c_{2\min}$ and $c_{2\max}$.***Step 3***: Train the FW-WSVM model with training set. The parameters *C* and δ vary as the particle travels.***Step 4***: Judge whether the desired accuracy is reached, if yes, output the optimal combined parameters *C* and δ of FW-WSVM model, and turn to Step 6;otherwise turn to Step 5 and continue iterating.***Step 5***: Update the parameters.
***substep 1***: Update pbest by comparing the current fitness value of the particle with its individual history optimal value.***substep 2***: Update gbest by comparing the current fitness value of particles with the global optimal value of population.***substep 3***: Update the particle velocity according to Equation (12).***substep 4***: Update particle position are updated according to Equation (13).
***Step 6***: Substitute *C* and δ into FW-WSVM, train the model by training set and output the trained model.

## 3. Research Design

### 3.1. Input Variable Selection

Let *S* be the dataset, which is described as follows:

(18) $$S=\left[\begin{array}{ccccc} s_{1,open} & s_{1,low} & s_{1,high} & s_{1,close} & v_{1}\\ s_{2,open} & s_{2,low} & s_{2,high} & s_{2,close} & v_{2}\\ \vdots & \vdots & \vdots & \vdots & \vdots \\ s_{N,open} & s_{N,low} & s_{N,high} & s_{N,close} & v_{N} \end{array}\right]$$

where $s_{i,open}$, $s_{i,low}$, $s_{i,high}$, and $s_{i,close}$ denote the opening, lowest, highest and closing price, respectively, while $v_{i}$ is the trading volume for the ith(i=1,2,…,N) day. In the stock prediction problem, $s_{i,open}$, $s_{i,low}$, $s_{i,high}$, $s_{i,close}$ and $v_{i}$ provide very useful information and can usually be used as the input features. Recently, some research has shown that some technical indicators are more informative than prices [16,46,47,48,49]. Consequently, many important technical indicators will be taken into consideration as input features. These technical indicators are defined in Table 1. A brief description of each indicator is described here:

(i) Simple Moving Average (SMA)
  The SMA is a method of statistical analysis that averages prices within a certain period of time for smoothing data.
(ii) Exponential Moving Average (EMA)
  The EMA is a trend indicator. Its construction principle is to carry out a weighted arithmetic average on the price to judge the change trend for the price in the future.
(iii) Moving Average Convergence/Divergence (MACD)
  The MACD is a technical indicator that uses the aggregation and separation between the short-term exponential moving average and the long-term exponential moving average of the closing price to judge the time of buying and selling.
(iv) Average Transaction Price (ATP)
  The ATP is used to identify the average cost of a transaction and can provide more useful information.
(v) Relative Strength Index (RSI)
  The RSI is a technical curve made according to the ratio of the sum of the rise and fall in a certain period of time. It can reflect the prosperity of the market in a certain period of time.
(vi) Average True Range (ATR)
  The ATR measures volatility, taking into account any gaps in the price movement.
(vii) William’s %R Oscillator
  This is a momentum indicator that measures the overbought and oversold levels.
(viii) Stochastic %K %D
  This indicates the momentum of a stock and uses the current close price of the stock.
(ix) Average Directional Movement Index (ADX)
  The ADX can be used to help measure the overall strength of a trend.

After adding technical indicators, the new dataset *S* is reformulated as follows:

(19) $$\left[\begin{array}{c} \begin{array}{cccccccccc} s_{1,open} & s_{1,low} & s_{1,high} & s_{1,close} & v_{1} & TI_{1,1} & TI_{1,2} & TI_{1,3} & \cdots & TI_{1,m}\\ s_{2,open} & s_{2,low} & s_{2,high} & s_{2,close} & v_{2} & TI_{2,1} & TI_{2,2} & TI_{2,3} & \cdots & TI_{2,m}\\ \vdots & \vdots & \vdots & \vdots & \vdots & \vdots & \vdots & \vdots & \vdots & \vdots \\ s_{N,open} & s_{N,low} & s_{N,high} & s_{N,close} & v_{N} & TI_{N,1} & TI_{N,2} & TI_{N,3} & \cdots & TI_{N,m} \end{array} \end{array}\right]$$

where $TI_{i,j}$ represents the technical indicators.

### 3.2. Data Labeling

We used PLR to generate class labels. We first used PLR to generate turning points for the stock data. We then classified these turning points into three categories, which were valley turning points, peak turning points and other turning points. The turning point with a trough was marked as a buying point, while the peak was marked as a selling point, and the other points were marked as holding points. The holding point, buying point and selling point were numbered zero, one and two, respectively. After generating the class labels $y_{i}\in \left\{0,1,2\right\}$, the variables were scaled between 0 and +1 using the standard min-max formula. The dataset *S* was reformulated as follows:

(20) $$\left[\begin{array}{cc} \begin{array}{cccccccccc} {\hat{s}}_{1,open} & {\hat{s}}_{1,low} & {\hat{s}}_{1,high} & {\hat{s}}_{1,close} & {\hat{v}}_{1} & TI_{1,1} & TI_{1,2} & TI_{1,3} & \cdots & TI_{1,m}\\ {\hat{s}}_{2,open} & {\hat{s}}_{2,low} & {\hat{s}}_{2,high} & {\hat{s}}_{2,close} & {\hat{v}}_{2} & TI_{2,1} & TI_{2,2} & TI_{2,3} & \cdots & TI_{2,m}\\ \vdots & \vdots & \vdots & \vdots & \vdots & \vdots & \vdots & \vdots & \vdots & \vdots \\ {\hat{s}}_{N,open} & {\hat{s}}_{N,low} & {\hat{s}}_{N,high} & {\hat{s}}_{N,close} & {\hat{v}}_{N} & TI_{N,1} & TI_{N,2} & TI_{N,3} & \cdots & TI_{N,m} \end{array} & \begin{array}{c} y_{1}\\ y_{2}\\ \vdots \\ y_{N} \end{array} \end{array}\right]$$

### 3.3. Performance Measure

The most important goal of stock forecasting is to obtain high and stable profits. We used two investment strategies to objectively test the profitability of the forecast effect. Let $b_{stock}\left(i\right)$ be the balance number, $b_{money}\left(i\right)$ be the balance money, $Avg_{i}$ be the average price for day *i*, $v_{money}\left(i\right)$ be the total investment money until day *i* and $c_{buy}$ and $c_{sell}$ be the transaction cost rates of buying and selling, respectively.

***Strategy 1:*** This investment strategy is used to assess the benefits of having a large amount of funds.

(1) Buying strategy: If the forecast signal is a buying signal ($y_{i}=1$), then the investors spend $Need_{i}=100\times \left(Avg_{i}\times \left(1+c_{buy}\right)\right)$ money to buy 100 shares. After buying the stock, $b_{stock}\left(i\right)$, $v_{money}\left(i\right)$ and $b_{money}\left(i\right)$ are calculated as follows:

(21) $$b_{stock}\left(i\right)=\left\{\begin{array}{cc} 100 & \;\;\;\;if\;i=1\\ b_{stock}\left(i-1\right)+100 & \;\;\;\;if\;i>1 \end{array}\right.$$

(22) $$v_{money}\left(i\right)=\left\{\begin{array}{cc} 100\times (Avg_{1}\times \left(1+c_{buy}\right)) & \;\;\;\;if\;i=1\\ v_{money}\left(i-1\right)+\left(Need_{i}-b_{money}\left(i-1\right)\right) & \;\;\;\;if\;0\leq b_{money}\left(i-1\right)\leq Need_{i},i>1\\ v_{money}\left(i-1\right)+Need_{i} & \;\;\;\;if\;b_{money}\left(i-1\right)>Need_{i},i>1 \end{array}\right.$$

(23) $$b_{money}\left(i\right)=\left\{\begin{array}{cc} 0 & \;\;\;\;if\;i=1\\ b_{money}\left(i-1\right)-Need_{i} & \;\;\;\;if\;b_{money}\left(i-1\right)>Need_{i},i>1\\ 0 & \;\;\;\;if\;0\leq b_{money}\left(i-1\right)\leq Need_{i},i>1 \end{array}\right.$$

(2) Selling strategy: If the forecast signal is the selling signal ($y_{i}=2$) and $b_{stock}\left(i\right)>0$, then the investors sell all their shares. After selling the stock, $b_{money}\left(i\right)$ and $b_{stock}\left(i\right)$ are calculated according to Equations (24) and (25), respectively:

(24) $$b_{money}\left(i\right)=b_{money}\left(i-1\right)+b_{stock}\left(i\right)\times Avg_{i}\times \left(1-c_{sell}\right)$$

(25) $$b_{stock}\left(i\right)=0$$

***Strategy 2:*** This investment strategy is used to evaluate the return of having limited capital. The initial investment capital is set by $v_{money}\left(1\right)=b_{money}\left(1\right)=10,000$, expressed in CNY.

(1) Buying strategy: If $y_{i}=1$, then the investors spend the balance money to buy the number of shares $NeedBuy_{i}=\left\lfloor \frac{b_{money}\left(i\right)}{Need_{i}}\right\rfloor \times 100$, where ⌊x⌋ denotes the minimal positive integer that is less than *x*. After buying the stock, $b_{stock}\left(i\right)$, $v_{money}\left(i\right)$ and $b_{money}\left(i\right)$ are calculated as follows:

(26) $$b_{stock}\left(i\right)=\left\{\begin{array}{cc} NeedBuy_{i} & \;\;\;\;if\;i=1\\ b_{stock}\left(i-1\right)+NeedBuy_{i} & \;\;\;\;if\;i>1 \end{array}\right.$$

(27) $$v_{money}\left(i\right)=\left\{\begin{array}{cc} NeedBuy_{1}\times \left(Avg_{1}\times \left(1+c_{buy}\right)\right) & \;\;\;\;if\;i=1\\ v_{money}\left(i-1\right)+NeedBuy_{i}\times \left(Avg_{i}\times \left(1+c_{buy}\right)\right) & \;\;\;\;if\;b_{money}\left(i-1\right)>Need_{i},i>1 \end{array}\right.$$

(28) $$b_{money}\left(i\right)=\left\{\begin{array}{cc} 10000-NeedBuy_{1}\times \left(Avg_{1}\times \left(1+c_{buy}\right)\right) & \;\;\;\;if\;i=1\\ b_{money}\left(i-1\right)-NeedBuy_{i}\times \left(Avg_{i}\times \left(1+c_{buy}\right)\right) & \;\;\;\;if\;b_{money}\left(i-1\right)>Need_{i},i>1\\ b_{money}\left(i-1\right) & \;\;\;\;if\;0\leq b_{money}\left(i-1\right)\leq Need_{i},i>1 \end{array}\right.$$

(b) Selling strategy: If $y_{i}=2$ and $b_{stock}\left(i\right)>0$, then the investors always sell all their shares. Thus, $b_{money}\left(i\right)$ and $b_{stock}\left(i\right)$ are updated according to Equations (24) and (25), respectively.

At the end of an investment cycle, all shares must be sold on the last day. The profit of this strategy is calculated as follows:

(29) $$profit=\frac{b_{money}-v_{money}}{v_{money}}$$

## 4. Experimental Results and Analysis

### 4.1. Data Collection and Experimental Set-Up

To demonstrate the IPSO-FW-WSVM model’s performance, 25 stocks were randomly selected from the Shanghai and Shenzhen exchange markets. Table 2 describes the collected datasets. The time span for these stocks was from 1 June 2012 to 30 June 2014. The 25 stocks could be divided into 3 types according to the change rate of the closing price: uptrend, downtrend and steady trend. If the rate of change of the closing price from the starting day to the end of the test period was higher than 10%, then it was classified as an uptrend. If the rate of change was lower than 10%, then it was classified as a downtrend; otherwise, it was classified as a steady trend.

In the experiments, Matlab 2016B and libsvm-3.11 [50] are used. Table 3 depicts the parameters used in the Shanghai and Shenzhen stock exchanges, where the size of each training set was 220, the size of each testing set was 20, the transaction fee for buying and selling was 0.0015 and the parameter pct was 0.35. Table 4 depicts the parameters used in IPSO. Table 5 depicts the technical indicators used as input variables.

### 4.2. Experimental Results

In this section, we conduct some comparative experiments between the IPSO-FW-WSVM and PLR-ANN models to illustrate the effectiveness of the proposed model. We used the neural network toolbox in Matlab R2016B to construct the compared ANN model, which had a three-layered feedforward structure, and the number of neurons in the hidden layer was selected using searching through five-fold cross-validation.

Table 6, Table 7 and Table 8 list the comparison results between the IPSO-FW-WSVM and PLR-ANN models on testing set in the uptrend stocks, the steady trend stocks and the downtrend stocks.

In Table 6, it can be seen that the IPSO-FW-WSVM model outperformed the PLR-ANN model in all five uptrend stocks listed in both aspects of trading point prediction accuracy and trading profits. The average accuracy for the trading signal prediction of the IPSO-FW-WSVM method was 52.85% while it was 46.61% for the PLR-ANN model in all five stocks. Even in the accuracy of individual stocks, the IPSO-FW-WSVM method also performed better than the PLR-ANN method. In terms of profit, the IPSO-FW-WSVM model outperformed the PLR-ANN model for all stocks using strategy 1, with the average profit being 42.66%, which was 34.39 points higher than that for the PLR-ANN model. Meanwhile, the IPSO-FW-WSVM algorithm requires less capital and less time to purchase than the PLR-ANN method, which shows that our proposed method is effective when using strategy 1. In the transaction with strategy 2, the IPSO-FW-WSVM model stood out against the PLR-ANN for five stocks receiving higher profits, in which the average profit of the IPSO-FW-WSVM model was 63.87%, while it was 11.18% for the PLR-ANN. In this way, we can conclude that our proposed method was more effective in the uptrend category and achieved better profitability using different investment strategies.

Table 7, demonstrates the similar comparative performances results between the IPSO-FW-WSVM and PLR-ANN models for the steady trend stocks and those for the uptrend stocks. The prediction accuracy of the IPSO-FW-WSVM model was higher than that for the PLR-ANN model for all steady trend stocks, being 51.56% for the IPSO-FW-WSVM while it was 47.80% in the PLR-ANN. In the transaction with strategy 1, the average profit for the IPSO-FW-WSVM method was 19.23%, while it was 7.04% for the PLR-ANN method. In the transaction with strategy 2, the IPSO-FW-WSVM also performed better than the PLR-ANN in terms of profit making with the same investment fund, with the average profit for the IPSO-FW-WSVM being 26.91%, while it was 0.41% for the PLR-ANN. The table also shows that the IPSO-FW-WSVM model needed to invest less capital and less time for purchases than the PLR-ANN model. From the above analysis, we can see that our proposed IPSO-FW-WSVM method can get better profit with different transaction strategies in the steady trend.

The results in Table 8 show that the IPSO-FW-WSVM model outperformed the PLR-ANN method for the downtrend stocks. Therefore, our proposed method was more effective in the downtrend category.

**Figure 4:**
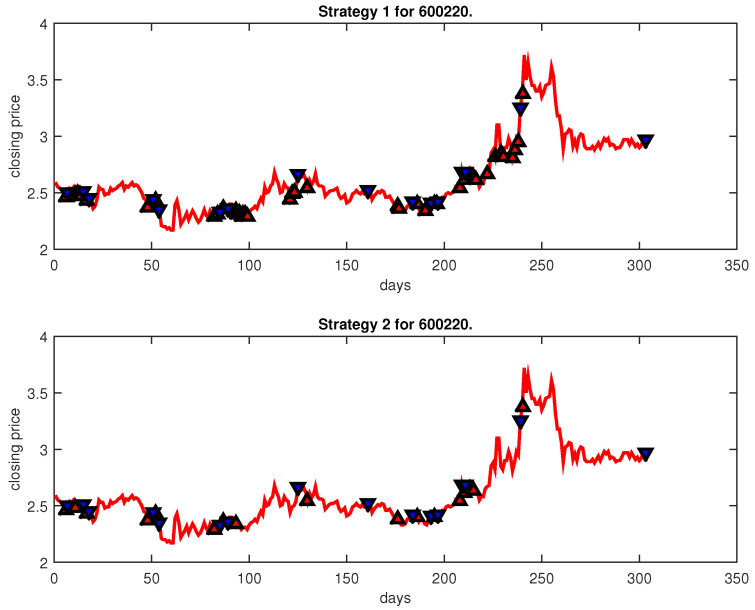
Trading signals for 600220 adopting IPSO-FW-WSVM.

**Figure 5:**
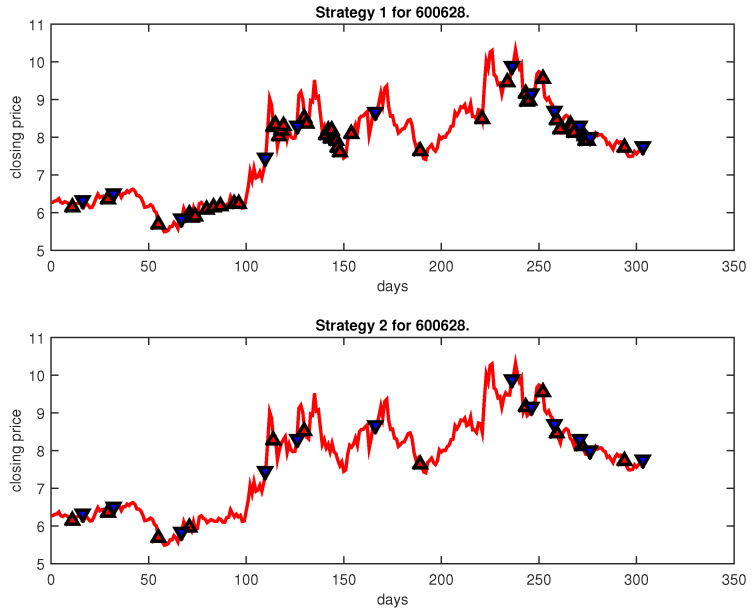
Trading signals for 600628 adopting IPSO-FW-WSVM.

**Figure 6:**
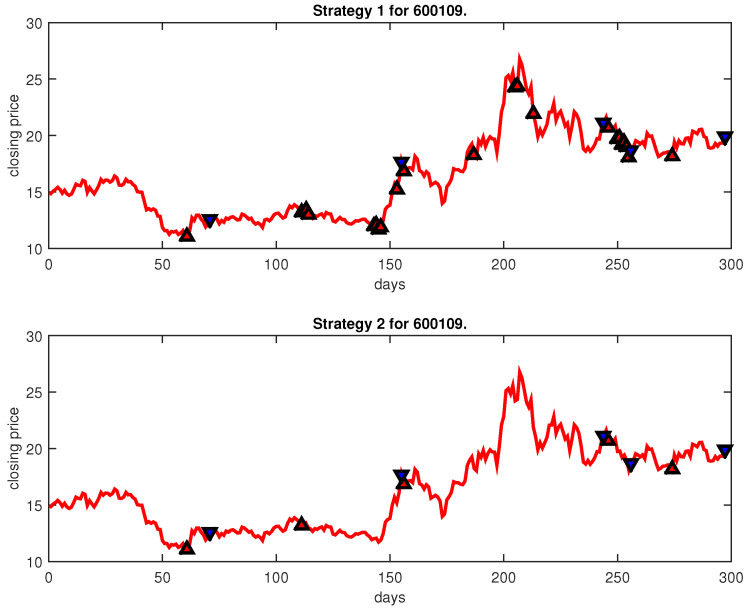
Trading signals for 600109 adopting IPSO-FW-WSVM.

**Figure 7:**
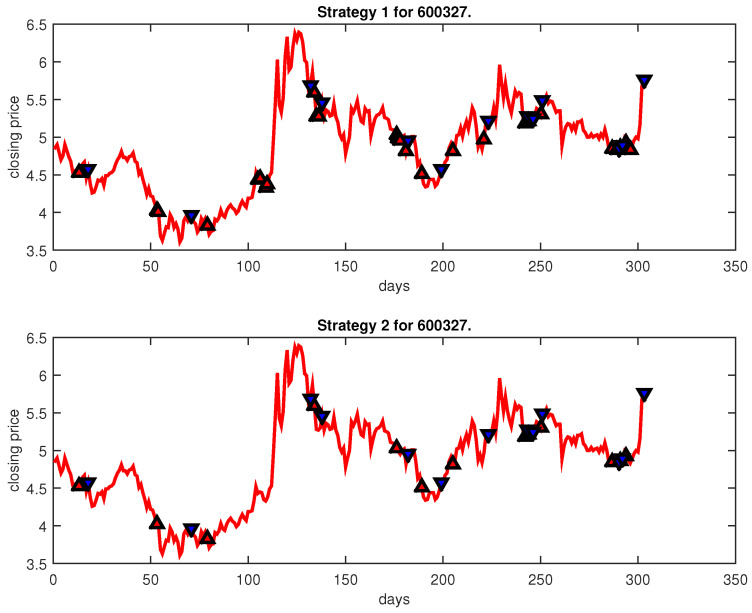
Trading signals for 600327 adopting IPSO-FW-WSVM.

**Figure 8:**
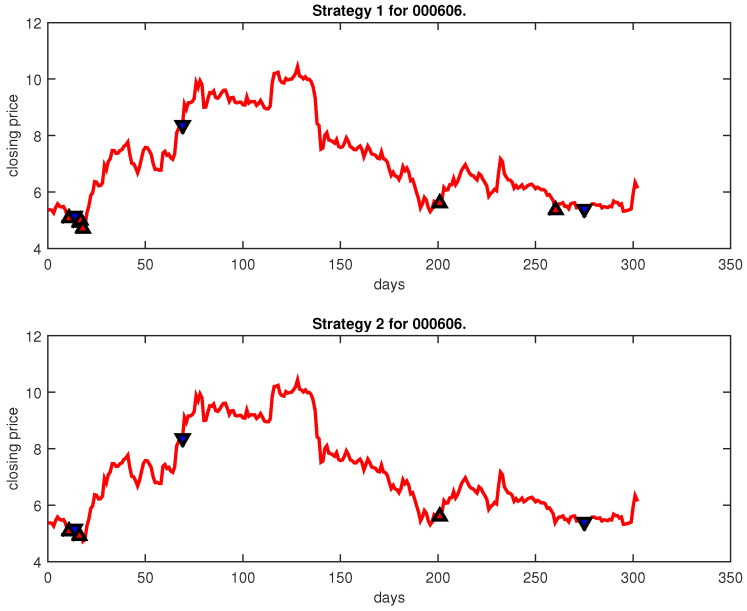
Trading signals for 000606 adopting IPSO-FW-WSVM.

**Figure 9:**
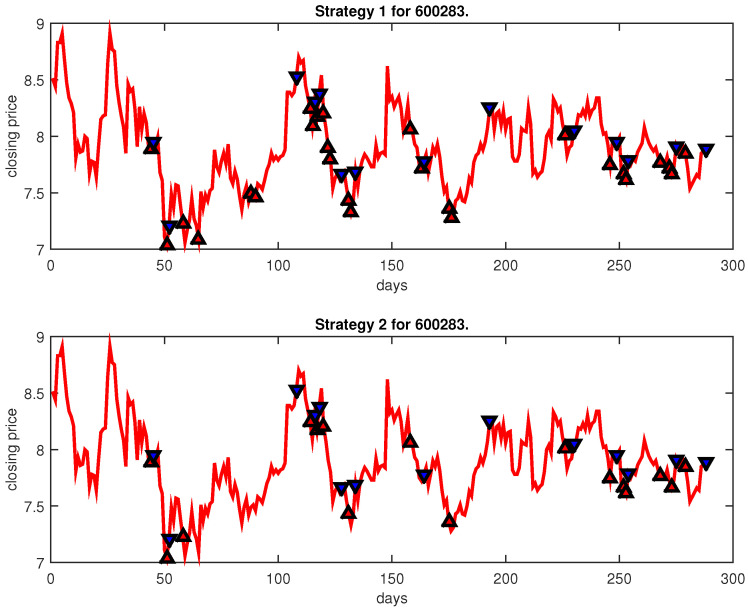
Trading signals for 600283 adopting IPSO-FW-WSVM.

**Figure 10:**
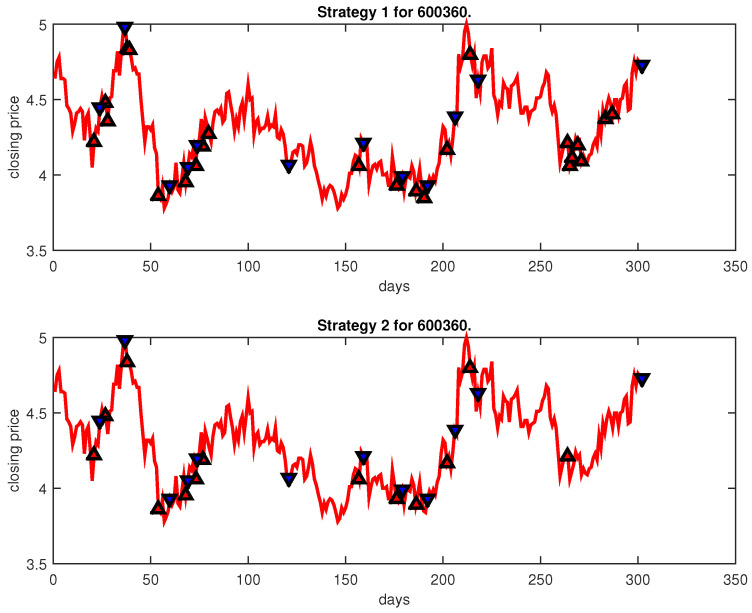
Trading signals for 600360 adopting IPSO-FW-WSVM.

**Figure 11:**
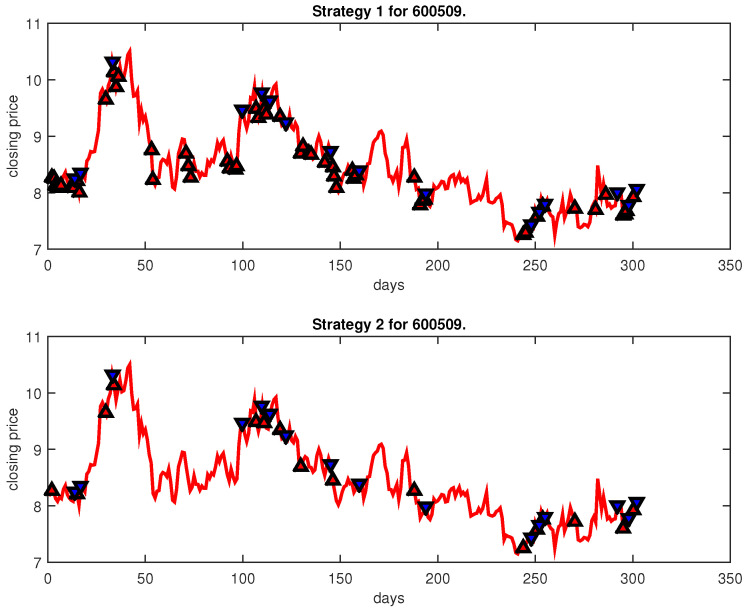
Trading signals for 600509 adopting IPSO-FW-WSVM.

**Figure 12:**
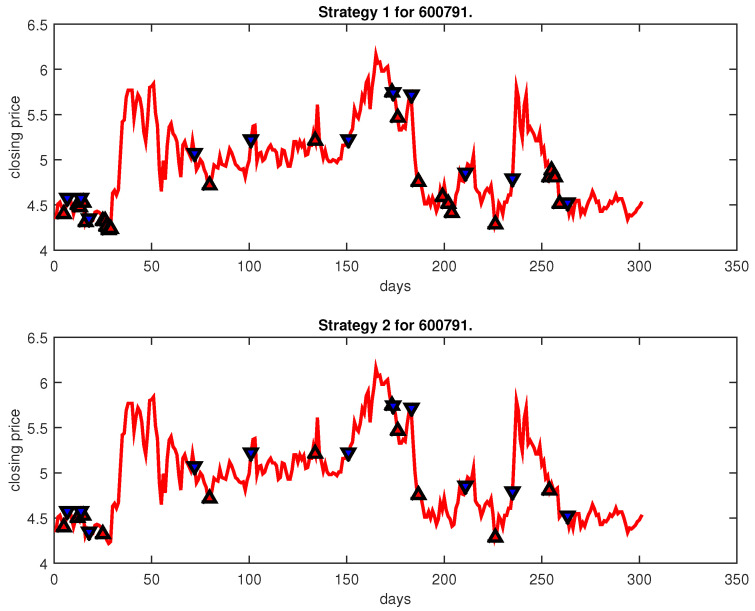
Trading signals for 600791 adopting IPSO-FW-WSVM.

**Figure 13:**
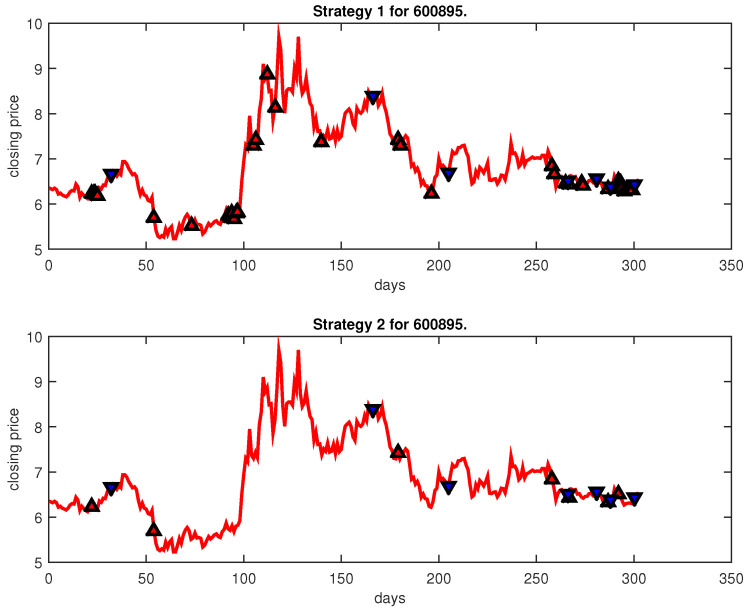
Trading signals for 600895 adopting IPSO-FW-WSVM.

**Figure 14:**
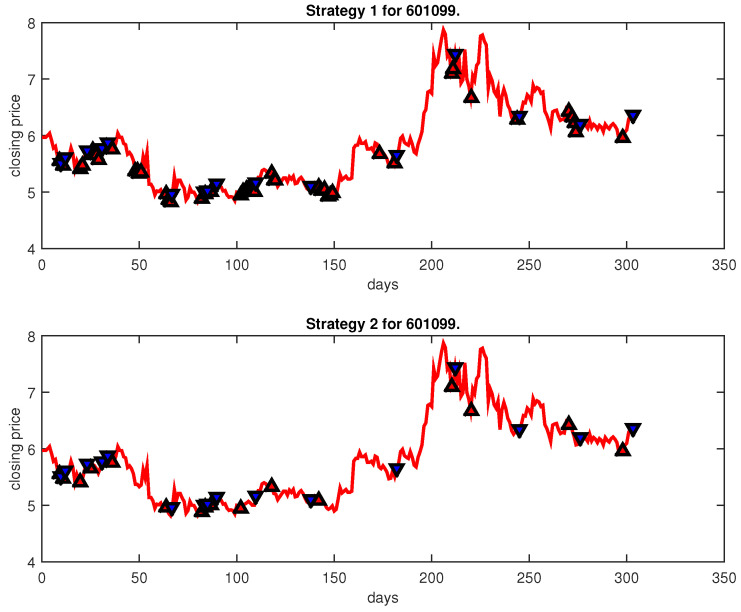
Trading signals for 601099 adopting IPSO-FW-WSVM.

**Figure 15:**
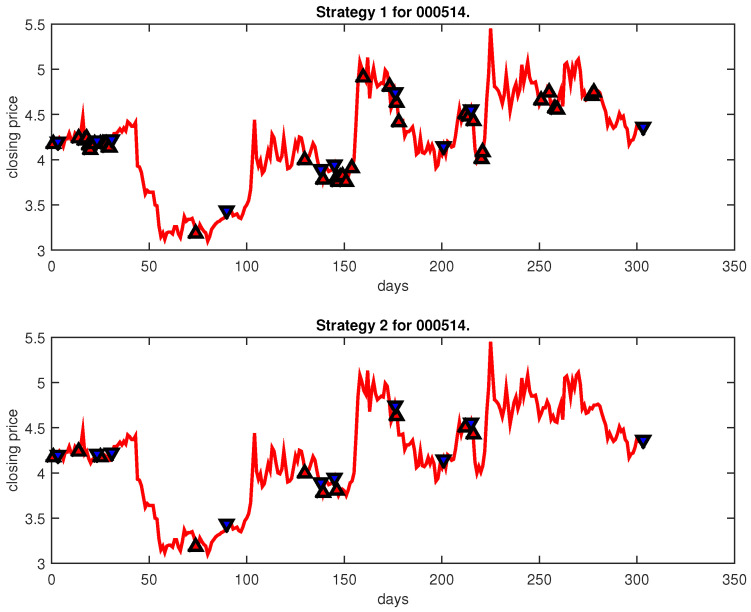
Trading signals for 000514 adopting IPSO-FW-WSVM.

**Figure 16:**
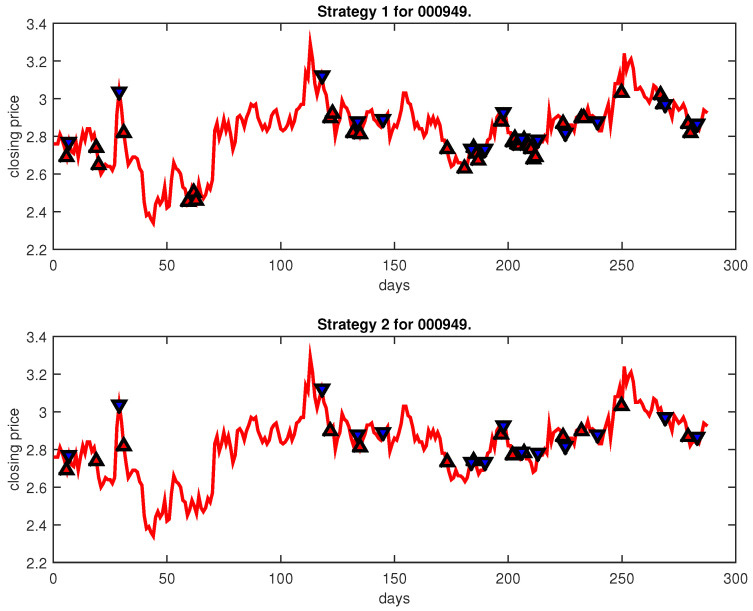
Trading signals for 000949 adopting IPSO-FW-WSVM.

**Figure 17:**
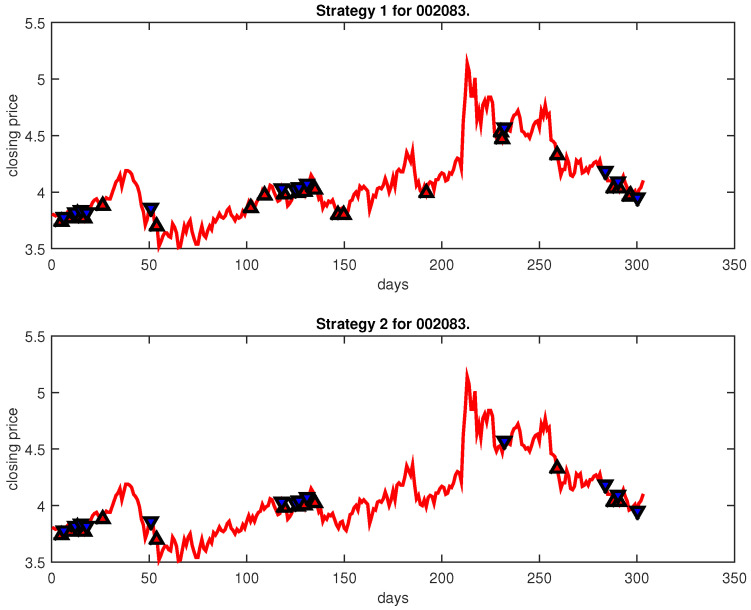
Trading signals for 002083 adopting IPSO-FW-WSVM.

**Figure 18:**
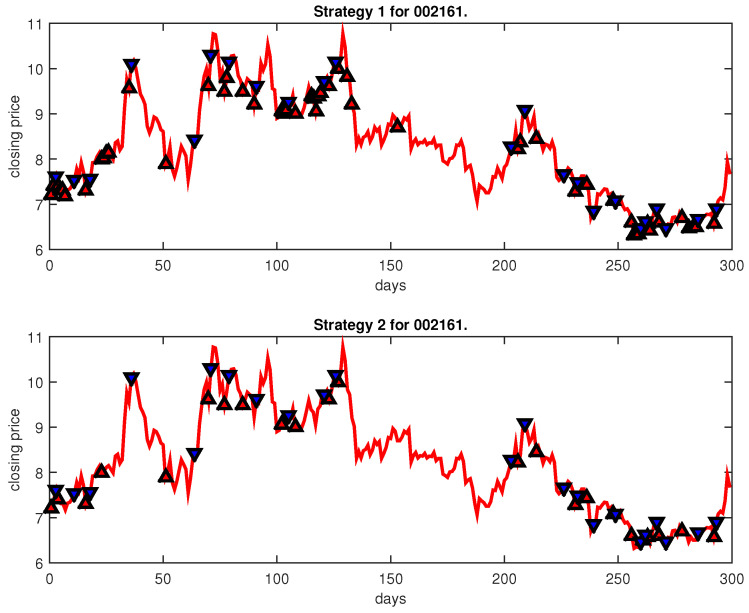
Trading signals for 002161 adopting IPSO-FW-WSVM.

**Figure 19:**
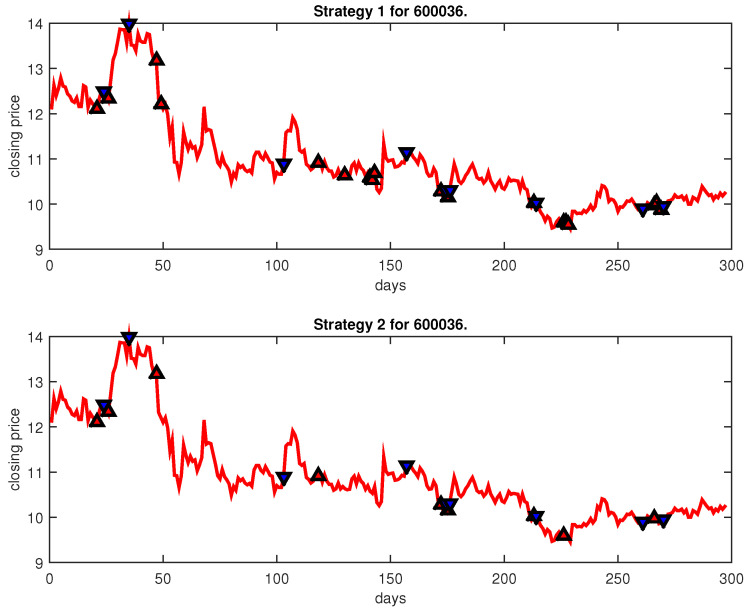
Trading signals for 600036 adopting IPSO-FW-WSVM.

**Figure 20:**
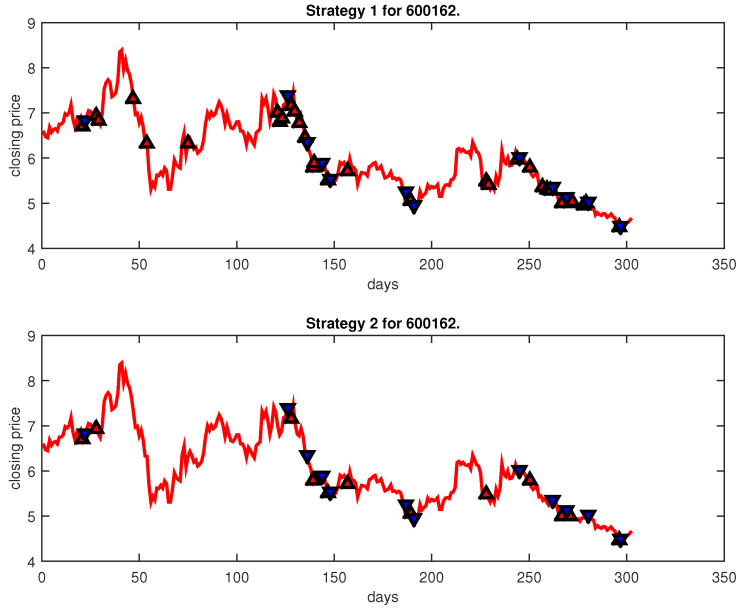
Trading signals for 600162 adopting IPSO-FW-WSVM.

**Figure 21:**
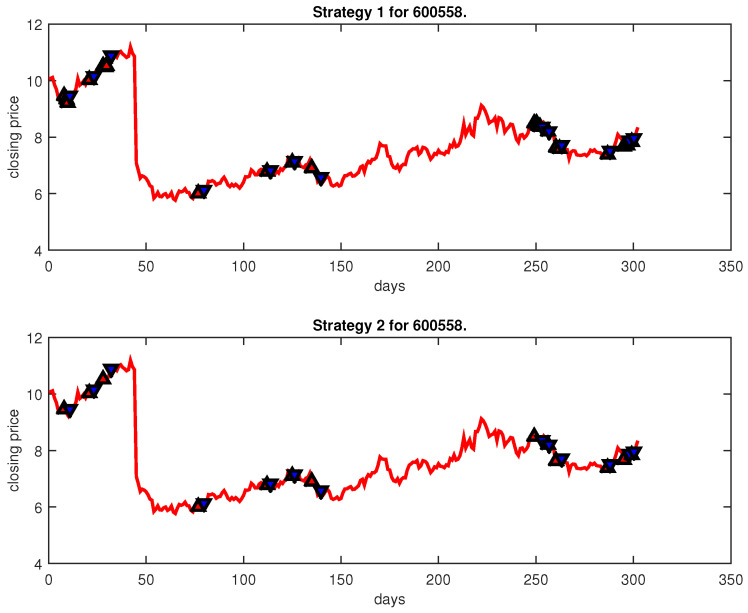
Trading signals for 600558 adopting IPSO-FW-WSVM.

**Figure 22:**
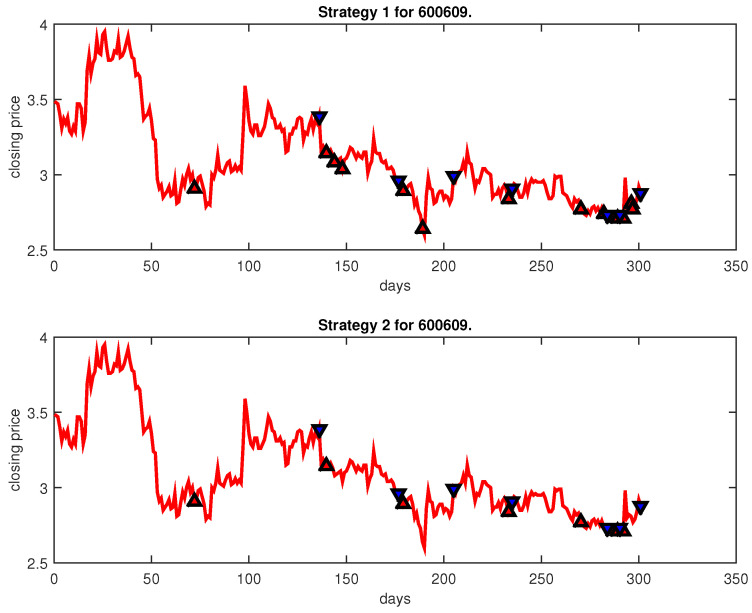
Trading signals for 600609 adopting IPSO-FW-WSVM.

**Figure 23:**
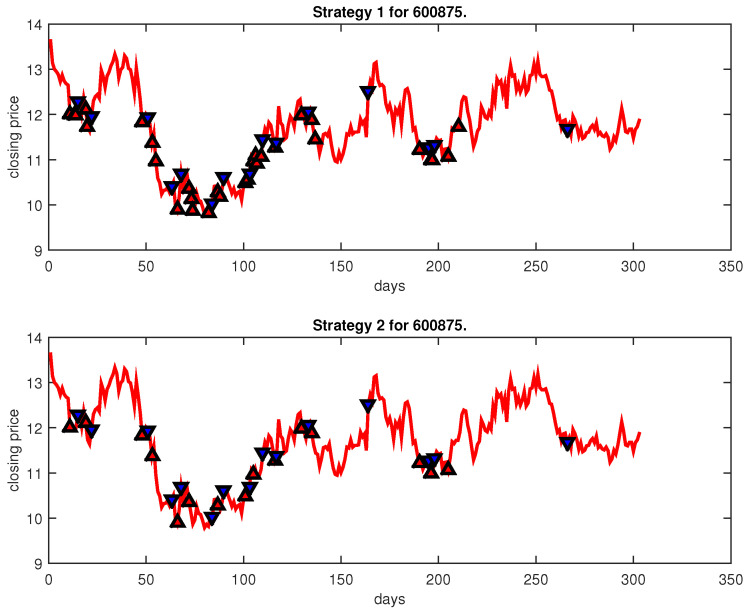
Trading signals for 600875 adopting IPSO-FW-WSVM.

**Figure 24:**
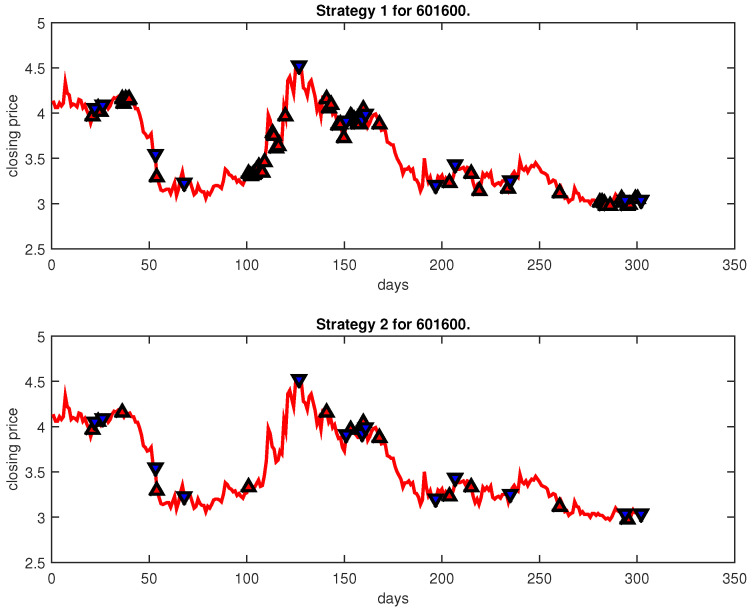
Trading signals for 601600 adopting IPSO-FW-WSVM.

**Figure 25:**
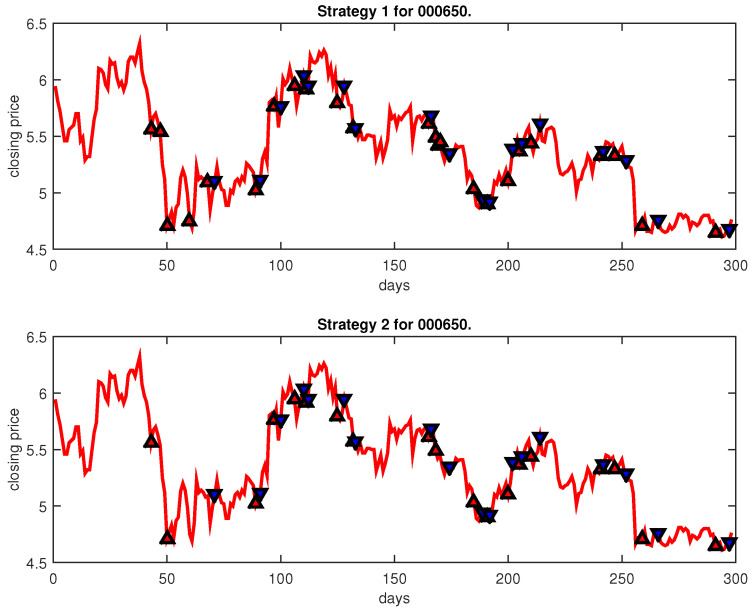
Trading signals for 000650 adopting IPSO-FW-WSVM.

**Figure 26:**
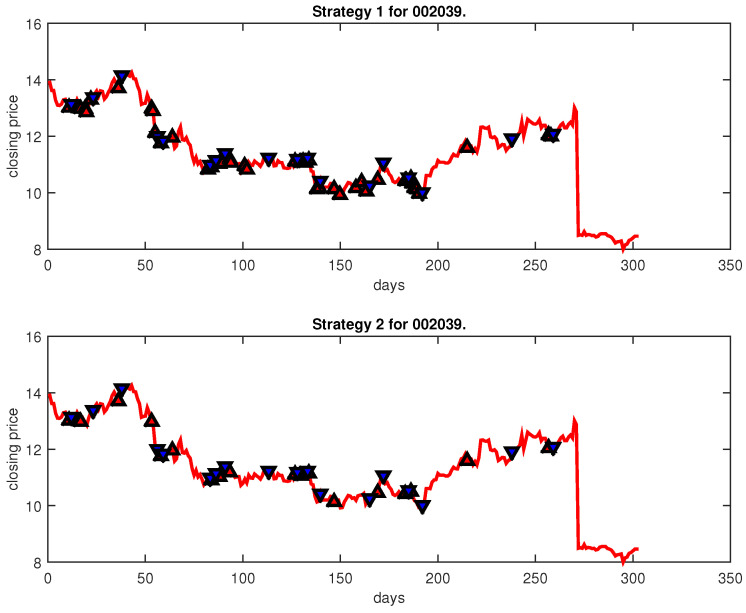
Trading signals for 002039 adopting IPSO-FW-WSVM.

**Figure 27:**
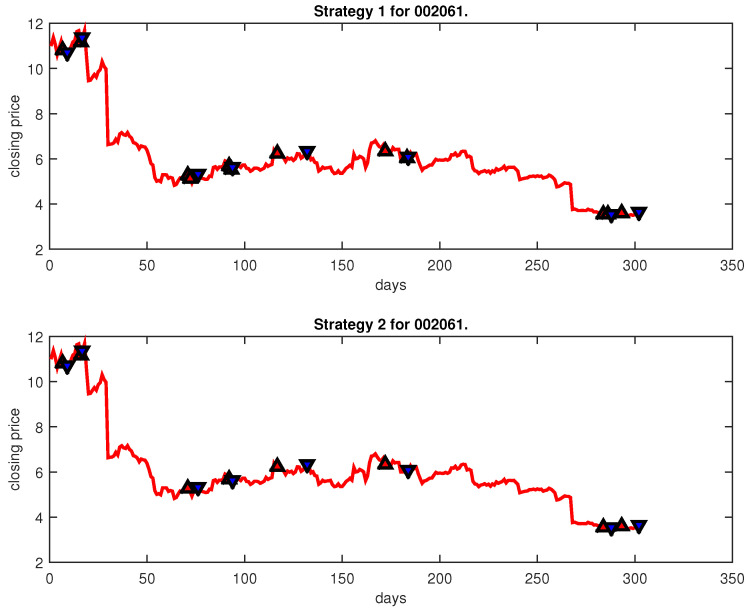
Trading signals for 002061 adopting IPSO-FW-WSVM.

**Figure 28:**
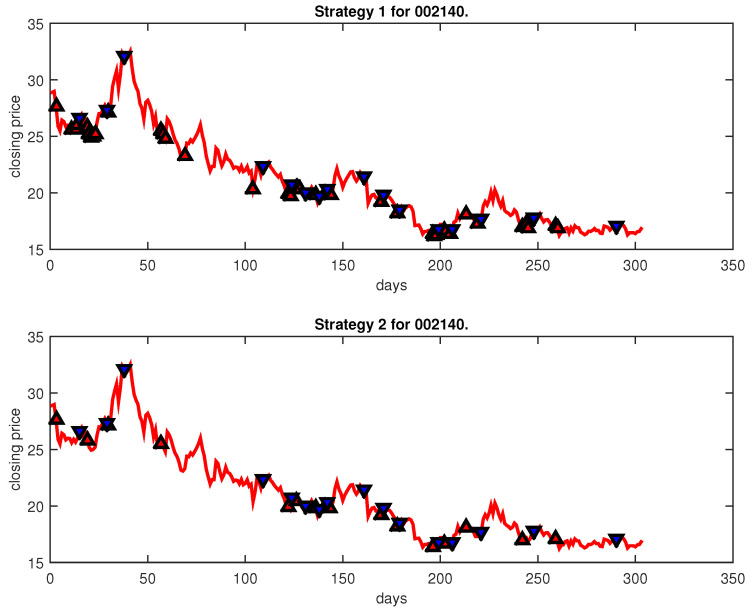
Trading signals for 002140 adopting IPSO-FW-WSVM.

Figure 4, Figure 5, Figure 6, Figure 7, Figure 8, Figure 9, Figure 10, Figure 11, Figure 12, Figure 13, Figure 14, Figure 15, Figure 16, Figure 17, Figure 18, Figure 19, Figure 20, Figure 21, Figure 22, Figure 23, Figure 24, Figure 25, Figure 26, Figure 27 and Figure 28 show the buying and selling signals when adopting the IPSO-FW-WSVM model on testing set for the uptrend stocks, the steady trend stocks and the downtrend stocks with two strategies. In the figures, the upper triangle (▲) denotes the buying signal, and the lower triangle (▼) denotes the selling signal. In the Figure 4, Figure 5, Figure 6, Figure 7, Figure 8, Figure 9, Figure 10, Figure 11, Figure 12, Figure 13, Figure 14, Figure 15, Figure 16, Figure 17, Figure 18, Figure 19, Figure 20, Figure 21, Figure 22, Figure 23, Figure 24, Figure 25, Figure 26, Figure 27 and Figure 28, it is shown that the buy and sell signals for both strategies were still far from optimal. We think this is reasonable. Due to the characteristics of the stock market, such as uncertainty, noise, non-stationarity and nonlinearity, stock trend forecasting is a very hard problem.

## 5. Conclusions

An amount of research has been carried out on the behavior of stock price movements. However, investors are more interested in obtaining profits. Therefore, trading decisions are more important than forecasting the stock prices themselves. This paper proposes a comprehensive and efficient trading signal forecasting framework. First, we applied PLR to decompose the historical data into different segments and model trading signal prediction as a three-class classification problem. Then, we trained the IPSO-FW-WSVM model using historical training data and compared the proposed method with a PLR-ANN on the stocks under different trends in Chinese stock exchange markets. The experiment’s results clearly illustrate that the IPSO-FW-WSVM model can obtain a significantly higher forecasting accuracy than the PLR-ANN model for stocks in three different tends. Moreover, the proposed framework can make a significant amount of profit with different trading strategies, and the proposed system is very effective at predicting future trading points. However, there are still some problems to be studied further. Although the algorithm we proposed has many advantages, which we mentioned above, it also has some disadvantages. For example, it is difficult to implement for large-scale training samples and sensitive to missing data. Future research work will involve designing better forecasting models to make the results more accurate. Future research will also explore other good investment strategies, since different investment strategies have a large effect on profits, and an unsuitable strategy can lead to poor returns despite the high forecasting performance.

## Figures and Tables

**Table 1:** Used technical indicators with formulae.

Technical Indicator	Formulae
1. Simple Moving Average (SMA)	$SMA_{i}\left(n\right)=\frac{1}{n}\sum_{j=\max\{1,i-n\}}^{i}s_{j,close}$
2. Exponential Moving Average (EMA)	$EMA_{i}\left(n\right)=\left\{\begin{array}{cc} s_{1,close} & if\;n=1\\ \frac{2}{n+1}*s_{i,close}\;+\frac{n-1}{n+1}*EMA_{i-1}\left(n\right) & if\;n>1 \end{array}\right.$
3. Moving Average Convergence /Divergence (MACD)	$\begin{array}{cc} & DIF_{i}\left(n\right)=EMA_{i}\left(n_{fast}\right)-EMA_{i}\left(n_{slow}\right)\\ & DEA_{i}\left(n\right)=\alpha *DEA_{i-1}\left(n\right)+\left(1-\alpha \right)*DIF_{i}\left(n\right)\\ & MACD_{i}\left(n\right)=2*\left(DIF_{i}\left(n\right)-DEA_{i}\left(n\right)\right) \end{array}$
4. Average Transaction Price (ATP)	$ATP_{i}=\frac{s_{i,money}}{vol_{i}}$ $s_{i,money}$ indicates the transaction money in one day
5. Relative Strength Index (RSI)	$RSI_{i}\left(n\right)=100-\frac{100}{1+EMA_{i}\left(n_{up}\right)/EMA_{i}(n_{down})}$ $EMA_{i}\left(n_{up}\right)$ is upward change, and $EMA_{i}\left(n_{down}\right)$ is downward change
6. Average True Range (ATR)	$ATR_{i}=EMA_{i}(max(s_{i,high}-s_{i,low},|s_{i,high}-s_{i,close}-1|,|s_{i,low}-s_{i-1,close}\left|)\right)$ |…| denotes the absolute value, and *n* is the input window length
7. William’s %R Oscillator	$Williams\_R_{i}=100*\left(s_{i,high}-s_{i,close}\right)/\left(s_{i,high}-s_{i,low}\right)$
8. Stochastic %K %D	$\begin{array}{cc} & \frac{\;K_{i}\left(n\right)=100*(s_{i,close}-mean_{n,low}/\left(mean_{n,high}-mean_{n,low}\right)}{\;\%D_{i}\left(n\right)=EMA_{3}\left(\%K_{i}\left(n\right)\right)} \end{array}$ $mean_{n,high}$ and $mean_{n,low}$ are the mean high and low prices in the last *n* days, respectively
9. Average Directional Movement Index (ADX)	$ADX_{i}\left(n\right)=\frac{ADX_{i-1}\left(n\right)*\left(N-1\right)+DX}{n}$

**Table 2:** Description of data samples.

Trend	Stock Code in Shanghai and Shenzhen Markets	Data Range
uptrend	600220, 600628, 600109, 600327, 000606	from 1 June 2012 to 30 June 2014
steady trend	600283, 600360, 600509, 600791, 600895 601099, 000514, 000949, 002083, 002161	from 1 June 2012 to 30 June 2014
downtrend	600036, 600162, 600558, 600609, 600875 601600, 000650, 002039, 002061, 002140	from 1 June 2012 to 30 June 2014

**Table 3:** Description of the parameters.

$L_{\mathrm{train}}$	$L_{\mathrm{test}}$	$c_{\mathrm{buy}}$	$c_{\mathrm{sell}}$	pct
220	20	0.0015	0.0015	0.35

**Table 4:** Description of parameters used in IPSO.

*n*	$T_{\max}$	$\omega_{\min}$	$\omega_{\max}$	$c_{1\min}$	$c_{1\max}$	$c_{2\min}$	$c_{2\max}$
10	100	0.9	0.3	0.5	2.5	0.5	2.5

**Table 5:** Technical indicators used as input variables.

No.	TI.	No.	TI.	No.	TI.	No.	TI.	No.	TI.	No.	TI.	No.	TI.
1	ATP	2	9K	3	9DIF	4	9DEA	5	14RSI	6	12ROC	7	Williams
8	5MA	9	10MA	10	5EMA	11	9D	12	9K	13	14ATR	14	14ADX

**Table 6:** The comparison results between IPSO-FW-WSVM and PLR-ANN models for stocks in the uptrend.

Stock Code	Method	Accuracy	Strategy 1				Strategy 2			
			$V_{\mathrm{money}}$	Profit (%)	$N_{\mathrm{buy}}$	$N_{\mathrm{sell}}$	$V_{\mathrm{money}}$	Profit (%)	$N_{\mathrm{buy}}$	$N_{\mathrm{sell}}$
600220	IPSO-FW-WSVM	54.79	2348.55	30.43	40	16	10,000	37.31	16	16
	PLR-ANN	50.50	3505.79	1.49	68	15	10,000	23.94	16	15
600628	IPSO-FW-WSVM	46.53	7717.44	27.89	44	12	10,000	50.97	12	12
	PLR-ANN	43.56	45,344.00	15.29	133	4	10,000	8.79	5	4
600109	IPSO-FW-WSVM	51.18	10,108.92	35.77	22	5	10,000	81.77	5	5
	PLR-ANN	45.12	29,094.58	18.85	99	13	10,000	−5.30	14	13
600327	IPSO-FW-WSVM	52.81	2153.97	49.19	27	13	10,000	86.05	13	13
	PLR-ANN	46.53	35,816.77	12.08	107	5	10,000	30.70	5	5
000606	IPSO-FW-WSVM	58.94	1461.73	70.00	6	3	10,000	63.25	3	3
	PLR-ANN	47.35	20,000.81	−6.36	82	10	10,000	−2.23	11	10
Average	IPSO-FW-WSVM	52.85	4758.12	42.66	27.80	9.80	10,000	63.87	9.80	9.80
	PLR-ANN	46.61	26,752.39	8.27	97.80	9.40	10,000	11.18	10.20	9.40

**Table 7:** The comparison results between IPSO-FW-WSVM and PLR-ANN models for stocks in the steady trend.

Stock Code	Method	Accuracy	Strategy 1				Strategy 2			
			$V_{\mathrm{money}}$	Profit (%)	$N_{\mathrm{buy}}$	$N_{\mathrm{sell}}$	$V_{\mathrm{money}}$	Profit (%)	$N_{\mathrm{buy}}$	$N_{\mathrm{sell}}$
600283	IPSO-FW-WSVM	48.96	2912.90	25.67	26	14	10,000	34.61	16	14
	PLR-ANN	45.14	21,431.51	17.45	95	7	10,000	−2.47	7	7
600360	IPSO-FW-WSVM	52.65	2977.73	11.03	23	12	10,000	15.17	13	12
	PLR-ANN	50.33	16,218.29	6.94	59	5	10,000	9.81	5	5
600509	IPSO-FW-WSVM	49.34	10489.10	10.76	53	16	10,000	8.36	16	16
	PLR-ANN	47.35	39,822.34	5.22	98	4	10,000	−16.35	4	4
600791	IPSO-FW-WSVM	53.16	2122.90	24.21	24	11	10,000	42.83	11	11
	PLR-ANN	46.51	23,834.09	0.36	94	5	10,000	−18.74	6	5
600895	IPSO-FW-WSVM	49.01	8335.45	29.82	35	7	10,000	33.13	7	7
	PLR-ANN	44.70	46,109.99	7.92	113	14	10,000	12.10	16	14
601099	IPSO-FW-WSVM	48.18	4160.28	9.55	48	16	10,000	11.83	17	16
	PLR-ANN	42.24	16,495.44	1.42	85	12	10,000	−9.05	12	12
000514	IPSO-FW-WSVM	55.12	3532.42	10.92	36	10	10,000	19.22	10	10
	PLR-ANN	48.18	9404.20	6.56	95	12	10,000	−12.75	12	12
000949	IPSO-FW-WSVM	52.61	1317.13	24.78	35	14	10,000	20.76	14	14
	PLR-ANN	50.52	8255.36	17.49	86	8	10,000	11.34	8	8
002083	IPSO-FW-WSVM	54.79	2385.71	13.71	23	13	10,000	23.64	13	13
	PLR-ANN	52.81	12,855.85	7.68	74	6	10,000	19.42	6	6
002161	IPSO-FW-WSVM	51.84	4235.11	31.87	50	23	10,000	59.50	23	23
	PLR-ANN	50.17	37,128.73	−0.60	86	4	10,000	10.77	4	4
Average	IPSO-FW-WSVM	51.56	4246.87	19.23	35.30	13.60	10,000	26.91	14.00	13.60
	PLR-ANN	47.80	23,155.58	7.04	88.50	7.70	10,000	0.41	8.00	7.70

**Table 8:** The comparison results between IPSO-FW-WSVM and PLR-ANN models for stocks in downtrend.

Stock Code	Method	Accuracy	Strategy 1				Strategy 2			
			$V_{\mathrm{money}}$	Profit (%)	$N_{\mathrm{buy}}$	$N_{\mathrm{sell}}$	$V_{\mathrm{money}}$	Profit (%)	$N_{\mathrm{buy}}$	$N_{\mathrm{sell}}$
600036	IPSO-FW-WSVM	51.18	5510.71	2.43	18	9	10,000	1.92	9	9
	PLR-ANN	49.83	37,849.97	2.11	79	6	10,000	−11.82	6	6
600162	IPSO-FW-WSVM	46.69	5434.19	5.84	31	12	10,000	−9.27	12	12
	PLR-ANN	42.72	40,799.91	−22.02	93	10	10,000	−40.07	11	10
600558	IPSO-FW-WSVM	49.67	3251.21	4.81	22	13	10,000	4.74	13	13
	PLR-ANN	44.70	33,107.92	1.96	102	8	10,000	−16.45	9	8
600609	IPSO-FW-WSVM	53.49	880.33	9.40	13	7	10,000	20.09	7	7
	PLR-ANN	50.17	14,745.39	1.10	67	4	10,000	−20.88	4	4
600875	IPSO-FW-WSVM	45.54	4327.49	12.37	30	15	10,000	19.70	15	15
	PLR-ANN	42.57	42,984.28	−8.28	98	10	10,000	−10.07	10	10
601600	IPSO-FW-WSVM	51.99	5194.73	20.26	53	13	10,000	−10.81	13	13
	PLR-ANN	43.05	11,981.77	−9.94	143	11	10,000	−25.50	11	11
000650	IPSO-FW-WSVM	51.68	2568.85	0.53	24	18	10,000	2.21	19	18
	PLR-ANN	47.99	33,281.13	−10.22	87	3	10,000	−14.08	3	3
002039	IPSO-FW-WSVM	53.97	5207.73	1.63	42	18	10,000	−6.31	18	18
	PLR-ANN	47.35	25,229.79	−22.27	69	9	10,000	−42.19	9	9
002061	IPSO-FW-WSVM	50.66	1202.87	1.84	12	8	10,000	−1.09	8	8
	PLR-ANN	43.38	23,466.05	−20.45	87	5	10,000	−66.88	6	5
002140	IPSO-FW-WSVM	49.50	12,493.64	13.49	38	16	10,000	20.99	16	16
	PLR-ANN	43.23	104,549.51	−21.36	127	10	10,000	−47.03	10	10
Average	IPSO-FW-WSVM	50.44	4607.18	7.26	28.30	12.90	10,000	4.22	13.00	12.90
	PLR-ANN	45.50	36,799.57	−10.94	95.20	7.60	10,000	−29.50	7.90	7.60

## Data Availability

Not applicable.
